# General cognitive decline does not account for older adults’ worse emotion recognition and theory of mind

**DOI:** 10.1038/s41598-022-10716-9

**Published:** 2022-04-26

**Authors:** Qiuyi Kong, Nicholas Currie, Kangning Du, Ted Ruffman

**Affiliations:** Department of Psychology, University of Otago, Dunedin, New Zealand

**Keywords:** Ageing, Psychology

## Abstract

Older adults have both worse general cognition and worse social cognition. A frequent suggestion is that worse social cognition is due to worse general cognition. However, previous studies have often provided contradictory evidence. The current study examined this issue with a more extensive battery of tasks for both forms of cognition. We gave 47 young and 40 older adults three tasks to assess general cognition (processing speed, working memory, fluid intelligence) and three tasks to assess their social cognition (emotion and theory-of-mind). Older adults did worse on all tasks and there were correlations between general and social cognition. Although working memory and fluid intelligence were unique predictors of performance on the Emotion Photos task and the Eyes task, Age Group was a unique predictor on all three social cognition tasks. Thus, there were relations between the two forms of cognition but older adults continued to do worse than young adults even after accounting for general cognition. We argue that this pattern of results is due to some overlap in brain areas mediating general and social cognition, but also independence, and with a differential rate of decline in brain areas dedicated to general cognition versus social cognition.

## Introduction

Aging is associated with worse emotion recognition (ER) relative to young adults^1,2^, and worse theory of mind (ToM)^3^. Collectively, we will refer to these abilities as *social cognition*. There have been different explanations given as to why older adults struggle on such tasks: (1) they have a preference for positive emotional stimuli (i.e., positivity bias) that makes it difficult to process negative emotions^4–6^, (2) they have difficulty with still photos because these stimuli lack ecological validity^7^, (3) their difficulty stems from a failure to look at the eyes where important affective information is yielded^8–10^, (4) their general cognitive decline makes it hard to identify emotions and mental states^11–14^, or (5) their brain decline in frontal and temporal areas results in a decline in social cognition^2,15^.

These views have previously been critiqued^15^. For instance, (a) older adults struggle with both negative *and* positive emotions and not merely negative emotions^2^; (b) they have difficulty with ecologically valid stimuli such as videos and not just still photos^1^, and their difficulty on still photo tasks is ecologically valid in that it correlates with (i) difficulty detecting lies^16^, (ii) difficulty detecting faux pas^17^, (iii) greater verbosity^18^, and (iv) more right-wing authoritarian attitudes such as, “A woman’s place is in the home”^19^; (c) they have difficulty with emotion bodies and voices^2^ when a failure to look at the eyes isn’t relevant. Thus, these explanations do not seem adequate, yet general cognitive decline and brain decline are known features of aging and cannot be ruled out so easily. Therefore, we consider the evidence for general cognitive decline below, and we return to the brain decline explanation in the “Discussion”.

### Does general cognitive decline explain worse emotion recognition in older adults?

Aging is accompanied by decline in three core cognitive abilities: processing speed, working memory, and fluid intelligence. Fluid intelligence refers to the ability to solve novel reasoning problems with minimal input from prior learning such as formal schooling. Working memory is utilized when a task places simultaneous demands on processing and storage (e.g., short-term memory). These abilities are inter-related in that fluid intelligence benefits from a faster processing speed and better working memory^20,21^, faster processing speed is linked to better working memory in older adults^22^, and processing speed, working memory and fluid intelligence all decline in old age^23,24^. Thus, to index age-related cognitive decline, measures of processing speed, working memory and fluid intelligence are all central. Given these age-related declines in cognition, researchers have speculated that general cognitive decline might underlie older adults’ worse social cognition. Indeed, there have now been several studies that have examined this question that we review below, beginning with ER in young and middle-aged adults.

#### Young and middle-aged adults

In order to examine the role of general cognition in *older adults*, we first consider the role of general cognition for ER in *young and middle-aged adults*. A number of studies indicate that the fluid and general intelligence of young and middle-aged adults is related to their ER. For instance, ER in young adults correlates with general measures of cognition tapping memory and fluid intelligence^25^. Likewise, in a study of young and middle-aged adults, ER was related to general intelligence (verbal ability, induction, sequential reasoning, quantitative reasoning)^26^.

These relations have been investigated more thoroughly in a recent meta-analysis^27^. Amongst young and middle-aged participants, finding there was a mean effect size of *r* = 0.19 between higher intelligence and better ER with similar effects on ER for crystallized IQ, fluid IQ, spatial ability, memory, and speed. They also directly compared those under 35 to those over 35 and found higher overall correlations in the older group (e.g., crystallized IQ: *r*_<35_ = 0.20, *r*_>35_ = 0.28; fluid IQ: *r*_<35_ = 0.18, *r*_>35_ = 0.25). Although crystallized IQ (e.g., vocabulary) correlated with ER, crystallized IQ generally maintains or improves with age^28^. Thus, it does not seem plausible that crystallized IQ underlies the age-related decrement in ER in older adults. Instead, given (a) the relation between general cognition and ER in young adults, and (b) the decline in general cognition in older adults, it seems plausible that (c) general cognitive decline might account for worse ER in older adults. Below, we consider relevant research.

#### Older adults

There are a number of studies that have obtained correlations between general cognition and ER, either in a sample of older adults, or over a group of young and older adults^29–38^. There are also some studies that have not obtained significant correlations^39–41^. The contradiction in findings is complicated further by the fact that there are differences in how researchers have examined correlations, sometimes grouping all participants regardless of age, and sometimes examining correlations just in older adults themselves. In the latter case, the correlations provide information about potential decline *only within the older adult group* itself, rather than across the entire age span. However, if one wants to know whether general cognitive decline explains differences between young and older adults’ social cognition, it would make more sense to compute correlations over all participants, not just older adults.

Furthermore, in order to understand the relation between general and social cognition, it is necessary to do more than examine correlations. A key question is whether age-related difficulties are maintained after controlling for general cognition. Once again, however, research addressing this question is inconsistent. For instance, some studies indicate that age differences exist even after controlling for general cognition^30,33,37,40–43^. Yet, there are other studies where (a) age effects are eliminated for at least *some* of the emotion tasks administered when controlling for general cognition^32,36,44,45^, (b) age differences *do not exist at all* after controlling for general cognition^31,46^, or (c) age effects are substantially reduced. For instance, in one study there were “strong reductions” in effect size^29,34,47^.

Further compounding the difficulty in interpreting previous findings, there are important differences in the way in which researchers have indexed general cognition by using tests of speed, working memory, or fluid intelligence, as well as the tasks used to measure each of these abilities. Whereas it might be more informative to include multiple tests tapping different aspects of general cognitive abilities, 9 of 14 studies that have examined whether age effects are independent of general cognition have included a *single task* to measure a *single* cognitive ability^29,34–37,41,44,46^. Likewise, 9 of 14 studies have included a single task to measure social cognition^29,31,32,34,43–47^. These studies do not provide a comprehensive test of the relation between general and social cognition. What is needed, and what we provide in the present study, is an analysis with multiple tests that get at different aspects of both general and social cognition.

Adding to the confusion, inconsistencies have arisen even when the same task has been used to index general cognition. For instance, consider the studies that have given just a matrices task as a measure of general cognition. After controlling for matrices performance, three studies found age differences in social cognition^27,34,35^, one didn’t^31^, two found age differences on some social tasks but not others^36,44^, and one found a substantially reduced difference^25^. Thus, even when (a) the same task—matrices—is used to index general cognition, (b) matrices is one of the primary ways of examining fluid intelligence^48^, and (c) fluid intelligence is one of the most consistent abilities to decline in old age^23^, results still seem inconsistent.

In sum, (a) general cognition and social cognition are multi-faceted but previous studies have usually used a single measure of each, and (b) when one such single measure (matrices) has been used, the link to social cognition is inconsistent. Therefore, a more conclusive test of the relation between general and social cognition requires the inclusion of multiple tasks to measure different cognitive abilities.

A similar argument applies to tasks examining ER because different tasks present different information processing demands. For instance, some have claimed that dynamic stimuli (e.g., videos) should offer older adults a more ecologically valid stimulus^8^, which should ease difficulty. Yet, others have found that, in general, dynamic stimuli place a heavier demand on information processing resources than static stimuli^48^. That is, a dynamic emotional stimulus places a heavier demand on processing speed, working memory and fluid intelligence compared to still photos, because of the need for quick processing and updating of information as the display changes from one expression to another. Consistent with this argument, dynamic stimuli are generally more difficult^48^, and a meta-analysis of emotion tasks indicates the same; older adults have more consistent difficulties across emotions when given emotion videos than when given emotion photos^1^.

For these reasons, in our study we used three tasks each to measure different aspects of both general and social cognition (for a total of six tasks). No study has provided such an extensive battery of tasks (with five studies using a total of two tasks, seven studies using a total of three tasks, and two using four).

### Does general cognitive decline explain worse theory of mind in older adults?

The previous section considered ER but a similar conflict in findings has arisen when researchers have examined the role of general cognition in older adults’ ToM. For instance, an early study examined the relation between processing speed, executive functioning (the Wisconsin Card Sorting Task) and ToM in young and older adults^11^. The ToM task (Strange Stories) required participants to explain unusual behaviour, and both processing speed and executive functioning correlated with ToM, although older adults were still worse than young adults after accounting for general cognition. Subsequently, a review of this study and many others concluded that, “ToM may be observed to function independently from general cognition in aging, but further investigation is needed to confirm this point” (p. 32)^12^.

Since that time, subsequent studies have produced conflicting findings. For instance, one study gave participants aged 17–95 the Strange Stories task, along with a Stroop task, a working memory task, and an empathy scale^13^. Performance on the cognitive measures as well as empathy correlated with performance on the ToM task, and when entered into a regression, explained all of the variance in ToM performance, with age not explaining significant variance.

Another study varied the executive function (EF) demands of a ToM task and found that the task with high EF demands led to age differences, whereas the task with low EF demands produced no age differences^14^. However, they measured ToM with 20 false and 20 true belief tasks, and this is a likely shortcoming of this study in that these tasks are passed by 4- to 5-year-old children typically, and therefore, do not provide a sensitive measure of ToM. It is probable that failure occurs only because of waning attention over 40 trials rather than a lack of conceptual insight, and this likely explains the relation to EF in this study, whereas a more conceptually demanding ToM task might be unrelated to EF.

Other researchers have examined EF abilities on tasks such as Trail Making (tapping speed and fluid IQ), Backwards Digit Span (working memory), the Tower of London (planning, inhibition), and matrices (fluid IQ) in a group of young adults^49^. None of these tasks correlated with performance on the Eyes task (judging complex emotions and mental states in the eyes). On the Strange stories task, only matrices correlated, but there was no older group to determine whether age differences still existed.

Still another study examined the Eyes task, along with aspects of executive function in a group of older adults^50^. Block Design and inhibitory ability correlated with ToM, but there was no younger age group to determine whether any potential older adult disadvantage on the Eyes task would remain significant after accounting for general cognition.

In sum, research presents a rather confusing picture. Sometimes general cognition correlates with ToM and sometimes it doesn’t. If general cognition correlates, sometimes age differences persist when controlling for general cognition, and sometimes they don’t.

### Present study

Previous studies of the relation between general cognition and ER have usually used one task that measures a single cognitive ability, with studies having obtained inconsistent findings. Inconsistency is also the norm when one narrows the lens to look only at studies employing a single cognitive task such as matrices. Yet there are multiple cognitive skills that are relevant to social cognition, with different social cognition tasks having different cognitive demands. In an effort to provide a more comprehensive index of cognitive ability, we used three tasks, all of which measured a different cognitive skill, and with all skills (1) thought to be central to cognitive decline: speed of processing, working memory, and fluid IQ^6^, (2) to be related to each other^20–24^, and (3) to decline in old age^20–24^.

Further, in a deliberate effort to vary the cognitive demands of the tasks tapping social cognition, we also included three social tasks: the Eyes task, an Emotion Photos task, and an Emotion Morph task. The Eyes task is typically thought of as a ToM measure, although it has more recently been argued that it is better construed as a test of complex emotions^51^. Analysis of the 36 items indicates that, perhaps 9 unambiguously measure emotion (e.g., despondent), 13 have an emotional valence (e.g., insisting), and 14 are more straightforward cognitive items (e.g., preoccupied). It might, therefore, represent something of a cross between a measure of more complex emotions and mental states, but can be construed as a more demanding task than classic ER tasks, which examine only basic emotions (anger, sadness, fear, disgust, surprise, and happiness). We used the Eyes task rather than other typical ToM measures, such as the Strange Stories task, because it is a highly researched task, and like the two emotion tasks, relies on processing of facial information (rather than inferring intentions from textual information). The Emotion Photos task is a standard ER task in which we presented still photos of the face expressing basic emotions. The Emotion Morph task presented dynamic stimuli, with emotion faces that gradually morphed from one basic emotion into a different basic emotion, and the participant’s task was to identify the new emotion as quickly as possible.

These three tasks tapping social cognition had intentionally differing characteristics that allowed us to carefully examine the relation between general cognition and social cognition. Again, the Eyes task required an understanding of complex emotions and mental states, but used still photos. The Emotion Photos tasks examined basic emotions and presented still photos. Neither of these tasks had a time limit on responding or required integration of information over time. In contrast, the Emotion Morph task required both of these things. As stated above, the Emotion Morph task should place a heavier demand on processing speed, working memory and fluid intelligence because of the need for quick processing and updating of information as the display changes from one thing to another^44^. This is particularly so because the task was speeded, in that participants were instructed to identify the new emotion as quickly as possible. Thus, to take into account both speed and accuracy, we computed a “balanced integration score” that gave equal emphasis to each. Given that speed was integral to optimal performance, and speed is a core cognitive ability that declines with age, logic would have it that the Emotion Morph task should be more heavily correlated with general cognition than the Eyes or Emotion Photos tasks.

## Results

### Analysis strategy

We first compared young and older adults on the different tasks, then we examined correlations between age and task performance and ran partial correlations between variables after controlling for age. Finally, we used regression to examine whether age differences in task performance were present after controlling for performance on the cognitive measures.

### Age differences in task performance

Table 1 includes the descriptive statistics for the main tasks. The Balanced Integration Score for the Emotion Morph task places equal emphasis on speed and accuracy^52^. The social cognition composite was calculated by averaging the proportion correct across the three tasks tapping social cognition. On all tasks, young adults performed at a higher level than older adults as measured by *t*-test. Although reliabilities are modest, we note that this is expected given that items were chosen to index a range of difficulties.

**Table 1:** Descriptive statistics and reliability.

	*α*	Young adults		Older adults	
		*M*	*SD*	*M*	*SD*
Matrices	.68	13.15^b^	1.38	11.85^b^	1.97
Processing speed	–	6.27^c^	0.62	4.84^c^	0.82
Working memory	.66	6.23^a^	1.77	5.45^a^	1.60
Eyes task proportion	.59	0.744^b^	0.090	0.673^b^	0.129
Emotion Morph Proportion correct	.61	0.671^c^	0.192	0.493^c^	0.198
Emotion Morph reaction time	–	4.58^c^	0.51	5.28^c^	0.45
Emotion Morph balanced integration score	–	0.948^c^	0.808	− 1.114^c^	1.270
Emotion photos proportion	.54	0.750^a^	0.091	0.699^a^	0.110
Social cognition composite	–	0.721^c^	0.091	0.622^c^	0.121

Cronbach’s alpha measures the reliability of tasks. For each task, we compared young and older adults’ performance using *t*-tests, with young adults *always* having better performance. Superscripts indicate the level of significant difference: ^a^*p* < .05, ^b^*p* < .01, ^c^*p* < .001.

Table 2 includes the correlations between variables. To provide maximum clarity, we used three measures for the Emotion Morph task: the Balanced Integration Score, proportion correct, and reaction time. The Balanced Integration Score is the most comprehensive measure and also tended to correlate most consistently with the other measures, so we retained this measure for the regressions below. Working memory and matrices correlated with all three tasks tapping social cognition, and processing speed correlated with one task (Emotion Morph). The social cognition composite correlated with matrices and working memory but not processing speed. We treated age as a continuous variable in the correlations, and advancing age correlated with worse performance on every task. The three tasks tapping social cognition correlated with each other.

**Table 2:** Correlations between main variables in all participants.

	1	2	3	4	5	6	7	8	9
1. Age	–								
2. Matrices	− .456^c^	–							
3. Processing speed	− .745^c^	.448^c^	–						
4. Working memory	− .287^b^	.454^c^	.305^b^	–					
5. Emotion photos	− .281^b^	.338^b^	.096	.292^b^	–				
6. Emotion Morphs BIS	− .748^c^	.384^c^	.572^c^	.317^b^	.516^c^	–			
7. Emotion Morph prop	− .435^c^	.167	.213^a^	.174	.607^c^	.683^c^	–		
8. Emotion Morph RT	.615^c^	− .330^b^	− .599^c^	− .233^a^	− .102	− .733^c^	− .041	–	
9. Eyes task	− .326^b^	.309^b^	.117	.324^b^	.432^c^	.459^c^	.340^b^	− .271^a^	
10. Social cog compos	− .434^c^	.339^b^	.177	.329^b^	.850^c^	.738^c^	.811^c^	− .173	.738^c^

Age was treated as a continuous variable in the correlations. Emotion Morph BIS: Balanced Integration Score. Emotion Morph Prop: Proportion correct. Emotion Morph RT: reaction time. Social Cog Composite: the average proportion correct across three tasks tapping social cognition. ^a^*p* < .05, ^b^*p* < .01, ^c^*p* < .001. (All tests two-tailed).

Table 3 lists the partial correlations between variables after controlling for age. We found weaker correlations between general cognition and social cognition after partialling out age, with Matrices correlating with only one social cognitive task (Emotion photos), Working Memory correlating with two tasks (Emotion Photos and the Eyes task), Processing Speed correlating with none of the three tasks tapping social cognition, and the social cognition composite correlating with processing speed only.

**Table 3:** Partial correlations between main variables after controlling for age over all participants.

	1	2	3	4	5	6	7	8
1. Matrices	–							
2. Processing speed	.182	–						
3. Working memory	.380^c^	.143	–					
4. Emotion photos	.246^a^	− .177	.230^a^	–				
5. Emotion Morphs BIS	.072	.034	.161	.481^c^	–			
6. Emotion Morph prop	.016	− .205	.112	.618^c^	.641^c^	–		
7. Emotion Morph RT	− .071	− .269^a^	− .075	.093	− .522^c^	.320^b^	–	
8. Eyes task	.190	− .200	.254^a^	.376^c^	.343^b^	.295^b^	− .095	
9. Social cognition composite	.176	− .244^a^	.237^a^	.842c	.612^c^	.800^c^	.133	.701^c^

Emotion Morph BIS: Balanced Integration Score. Emotion Morph Prop: Proportion correct. Emotion Morph RT: reaction time. Social Cognition: the average proportion correct across the three tasks tapping social cognition. ^a^*p* < .05, ^b^*p* < .01, ^c^*p* < .001. (All tests two-tailed).

We then used regression to examine whether the age group difference on each of the tasks tapping social cognition was still significant after controlling for general cognition. We entered Age Group as a dichotomous variable in the regression with participants divided into young and older age group. First, we examined the Emotion Morph BIS as the dependent variable, and included two steps in the regression, entering Age Group in the first step, and then the three general cognition tasks in the second step. Even though not all general cognition tasks were significant correlates of the tasks tapping social cognition, we entered all three in the second step to comprehensively control for cognition. Table 4 includes the results of this analysis for the Emotion Morph task. Recall that we posited that the Emotion Morph task should place the heaviest demands on general cognition because of the need for quick processing, yet only Age Group predicted unique variance.

**Table 4:** Regression predicting emotion Morph balanced integration score (speed + accuracy).

	*B*	*Beta*	*t*	*p*	Δ*R*^2^
Step 1					.497
Age group	− 2.062	− .705	− 9.17	< .001	
Step 2					.035
Age group	− 1.735	− .593	− 5.52	< .001	
Matrices	.063	.076	0.84	.405	
Processing speed	.114	.079	0.70	.486	
Working memory	.104	.123	1.44	.153	

Δ*R*^2^ represents the additional variance accounted for by all the variables in a step. Regression statistics given with relevant variables at a step in the prediction equation (i.e., Age Group at Step 1, and all four variables at Step 2). Age group was treated as a dichotomous variable (young vs. older adults) in the regression.

When Emotion Photos was the dependent variable (Table 5), Age Group, Matrices and Processing Speed were all significant predictors. When the Eyes task was the dependent variable (Table 6), Age Group, Speed and Working Memory were all significant predictors. When the Social Cognition Composite was the dependent variable (Table 7), Age Group and Speed were significant predictors. To summarize, for all three tasks measuring social cognition, Age Group continued to predict social cognition after accounting for general cognition, with processing speed predicting unique variance twice, and working memory and matrices predicting unique variance once each.

**Table 5:** Regression predicting emotion photos performance.

	*B*	*Beta*	*t*	*p*	Δ*R*^2^
Step 1					.061
Age group	− .050	− .247	− 2.35	.021	
Step 2					.139
Age group	− .068	− .332	− 2.36	.021	
Matrices	.016	.275	2.30	.024	
Processing speed	− .032	− .321	− 2.18	.032	
Working memory	.011	.190	1.70	.093	

Δ*R*^2^ represents the additional variance counted for by all the variables in a step. Regression statistics given with relevant variables at a step in the prediction equation. Age group was treated as a dichotomous variable (young vs. older adults) in the regression.

**Table 6:** Regression predicting eyes task performance.

	*B*	*Beta*	*t*	*p*	Δ*R*^2^
Step 1					.097
Age group	− .2.56	− .312	− 3.02	.003	
Step 2					.138
Age group	− 3.60	− .438	− 3.18	.002	
Matrices	0.46	.199	1.70	.092	
Processing speed	− 1.45	− .357	− 2.48	.015	
Working memory	0.58	.243	2.22	.029	

Δ*R*^2^ represents the additional variance counted for by all the variables in a step. Regression statistics given with relevant variables at a step in the prediction equation. Age group was treated as a dichotomous variable (young vs. older adults) in the regression.

**Table 7:** Regression predicting social understanding composite.

	*B*	*Beta*	*t*	*p*	Δ*R*^2^
Step 1					.183
Age group	− 0.10	− .428	− 4.42	< .001	
Step 2					.100
Age group	− 0.13	− .564	− 4.28	< .001	
Matrices	0.01	.156	1.73	.172	
Processing speed	− 0.04	− .336	− 2.54	.018	
Working memory	0.01	.202	2.16	.060	

Δ*R*^2^ represents the additional variance counted for by all the variables in a step. Regression statistics given with relevant variables at a step in the prediction equation. Age group was treated as a dichotomous variable (young vs. older adults) in the regression.

We reran all the analyses after excluding the older adults under 60 years (leaving 34 older adults) and obtained the same patterns in our results (i.e., age differences in all variables remained significant, correlations of interest were still significant, and Age Group continued to predict social cognition after accounting for general cognition in the regressions). Therefore, we only report findings with all participants included.

## Discussion

Older adults typically do worse on tests of both general and social cognition, leading to the suggestion that worse general cognition underlies worse social cognition. Yet, previous research is equivocal. General cognition sometimes correlates with social cognition and sometimes age differences are eliminated after accounting for general cognition. However, sometimes general cognition doesn’t correlate and age differences in social cognition persist.

One of the difficulties in interpreting previous findings is that the studies have usually examined only one of the three core cognitive abilities (processing speed, working memory, and fluid IQ) at a time. Indeed, 9 of 14 studies that have provided information on these questions have included a single task to measure general cognition, and 9 of 14 have, likewise, provided a single task to measure emotion recognition. It seems likely that better coverage of both general cognition and social cognition in an individual study would provide more comprehensive information as to their relation. Further, it seems important to choose tasks with varying cognitive demands; if these are central to performance, then relations with more cognitively demanding tasks should ensue. Thus, we examined this issue using a battery of tasks tapping both general cognition and social cognition.

Further, the three tasks measuring social cognition had varying cognitive demands. The Eyes task (which presented just the eyes and taps an understanding of complex emotions and mental states) used still photos. The Emotion Photos task (a classic test of ER) also used still photos, but examined just basic emotions. Neither the Eyes nor the Emotion Photos task had a time pressure or a need to integrate information over time. However, the Emotion Morphs task had both of these requirements. Following previous researchers^53^, we posited that the cognitive demands were greater for the Emotion Morph task given these considerations because it required quick processing and updating of information.

As anticipated, older adults did worse than young adults on all three measures of general cognition, and the same was true for social cognition. Further, the three measures of general cognition often correlated with social cognition. Working memory and matrices correlated with all three tasks tapping social cognition, and processing speed correlated with the Emotion Morph task. However, in the regressions, whereas age group was a unique predictor of performance on all three tasks tapping social cognition, general cognition was less consistently a unique predictor. For instance, consider the Emotion Morph task, which (a) was dynamic, thereby placing greater demands on speed, working memory and fluid intelligence, even when considering just the proportion correct component^53^, (b) but also had a speeded component that would further accentuate the cognitive demands, and (c) older adults were substantially worse than young adults with the correlation between the BIS and age equal to − 0.748 (meaning there was ample age variance to explain). Nevertheless, *none* of the three general cognition measures predicted unique variance on the Emotion Morph task. In other words, although all three cognitive tasks correlated with the Emotion Morph BIS, and there was a large age effect to explain, it could not be explained by a decline in general cognition. In contrast, and counter-intuitively, two of the three cognitive measures *did* predict unique variance on the Emotion Photos and Eyes tasks. This pattern of correlations suggests a somewhat random relation between general cognition and social cognition.

Overall, the findings can be summed up as follows: (a) in agreement with *some* past studies, general cognition sometimes correlates with social cognition and sometimes explains unique variance, but (b) despite this, there was an age effect in every single instance, such that older adults had worse social cognition on all three tasks after controlling for general cognition. Because previous studies had obtained somewhat inconsistent results, but had used few tasks to index general and social cognition, there was an ongoing need for further study to clarify findings.

A strength of the present study was that we examined both general cognition and social cognition more extensively than in prior research with no prior study giving participants such a large number and range of tasks to measure each. More specifically, we measured the three core components of general cognition—speed, working memory and fluid intelligence—known to deteriorate over age. Another strength is that we purposely varied the cognitive demands of the social cognition measures. Thus, our study provides a more thorough test of these relations. Although we didn’t measure some executive functions such as inhibitory ability, set shifting, or self-control, most social cognition measures (such as emotion recognition or the Eyes task) do not tax these skills to any obvious extent either. With such a large array of cognitive tasks possible, as well as tasks tapping social cognition (e.g., Strange Stories), no one study can do everything. Instead, we focussed on the most obvious cognitive correlates of the social cognitive tasks of interest.

At the outset, we outlined a number of theories as to why older adults have worse social cognition than young adults. The two theories that were difficult to rule out were general cognitive decline and brain decline. In fact, these two theories can be related at a deeper level because brain decline ultimately explains general cognitive decline. Thus, in our view, the most likely explanation is that some of the brain areas that mediate general cognition also mediate social cognition, yet with some distinction between these brain areas as well. For instance, working memory and fluid intelligence are thought to be primarily mediated by the dorsolateral prefrontal cortex^54^ as well as generalized changes in white matter^55^. Speed of processing is thought to be due to generalized changes in grey and white matter, including in frontal areas^56^.

As for social cognition, there is some overlap in that there is also involvement of the dorsolateral prefrontal cortex^57^, yet there is also independence, with evidence that ER relies on regions such as the orbitfrontal cortex, the anterior cingulate cortex and temporal lobes^2,4^. Similarly, the Eyes task engages frontal and temporal brain regions^58,59^. There will be some degree of overlap in the rate of decline in all brain areas, depending on factors such as diet and exercise^60^. Indeed, research shows that older adults with a healthier diet have relatively preserved general cognition *and* social cognition on a task tapping understanding of social gaffes, providing support for the brain decline hypothesis, and also, for a practical means of offsetting brain decline^61^. However, there is also evidence that these regions also undergo somewhat different rates of decline^62,63^. Thus, an analysis of brain regions and brain decline predicts both the overlap we obtained (i.e., the correlations between general cognition and social cognition), and the independence of these two abilities that we obtained.

In sum, we found that although aspects of general cognition correlate with success on tasks tapping social cognition, they do not fully explain age-related difficulties. Instead, brain decline might explain both the correlations between the two forms of cognition, with the overlapping brain areas underpinning associations between general cognition and social cognition, and their independence, resulting from the involvement of independent brain areas. For this reason, older adults continued to have difficulty on social cognitive tasks even after controlling for three core cognitive abilities known to decline over age.

## Method

### Participants

There were 47 young adults (9 males, 37 females, and 1 non-binary) ranging from 18 to 29 years old (M = 19.62 years, SD = 1.68) and 40 older adults (11 males and 29 females) ranging from 54 to 92 years old (M = 67.20 years, SD = 8.67). The young adults were university students who participated as part of their psychology courses, and the older adults were recruited through a university database and paid a nominal fee for travel expenses.

The undergraduates were all at a similar stage of their education. For older adults, we coded education on a 1 to 6 scale (1: primary school, 2: some high school, 3: high school diploma, 4: community college or polytechnic, 5: university undergraduate, 6: university post-graduate). Older adults’ education ranged from 1 to 6 with a mean of 2.59. To examine older adults’ cognitive health we gave them the Mini-Mental State Examination^64^. The maximum score a participant could receive was 30, with a score of 24 considered acceptable for a group with varying educational attainment. The mean score was 27.88, with a range of 24 to 30.

Participants received either course credit or travel money for their participation. Informed consent was provided by all participants. We carried out a power analysis using G*Power^65^ to determine the power for detecting age group differences using a *t*-test, as well as significant correlations, with the following constraints: two tails (allowing for either young or older adults to have better performance) and allowing for a moderate effect size (*d* = 0.30). The required sample size was 82. Thus, our sample of 87 should have been adequate to detect age group differences.

### Materials and procedure

Participants were tested in a quiet room of the laboratory at the Psychology Department. We randomized the tasks in five orders for each age group and then randomly assigned one order to each participant when administering the tasks. In all orders for older adults, we always gave the Mini-Mental State Examination first.

#### Matrices

The stimuli were pictures from the Wechsler Adult Intelligence Scale (WAIS-IV)^66^. The test is designed to measure visual processing and abstract spatial perception (i.e., fluid intelligence). Fourteen different matrices were used in the present experiment, which included two practice trials and 12 test trials. We selected item 3–14 from the original test to cover a full range of difficulty levels and avoid a ceiling effect. For each item, participants viewed an array of pictures with one missing square. Participants were told to select the picture that fitted the array from the five options presented below the picture, with the total score used in analyses.

#### Processing speed

We recorded the number of clicks a participant could make with the index finger of the dominant hand on a mouse during 5- and 20-s trials. The test score is the average number of clicks per second across the two trials. Essentially, this test is identical to a finger-tapping test, which requires participants to press a button/lever as rapidly as possible. The finger-tapping test is a traditional neuropsychological measure of processing speed adapted from the Halstead-Reitan Battery^67^. As there are only trivial differences between mouse clicking and finger tapping, we adapted mouse clicking test to measure processing speed for its easier administration.

#### Working memory

We used a backwards digit span test^66^ to examine working memory. Participants were asked to verbally recall the numbers in the reverse order they heard them. There were eight trials, consisting of two items each of three digits, four digits, five digits and six digits. On each trial the numbers were spoken by the experimenter at a rate of one number per second and participants were allowed to hear the set of numbers twice before giving their answer. Correct performance required the participant to repeat all digits in the correct (reversed) order.

#### Emotion Morph

The stimuli were black-and-white images selected from the Facial Expressions of Emotion Test^68^, which were manipulated to create six-second videos in which the face morphed from one emotion to another. In total there were 12 items, with two items (final emotion) for each of the six basic emotions. On each trial, the face started by expressing one emotion and morphed into one of the other five basic emotions. Participants were asked to stop the array when they could identify the new emotion, with the six basic emotions given on an answer sheet. The stimuli were created using MorphX^69^. In the descriptive statistics, we report proportion correct, reaction time, and a Balanced Integration Score^52^. In the subsequent analyses, we used the BIS given that both speed and accuracy are relevant to task success.

#### Still emotion recognition

The stimuli for this task were still images selected from the Emotion Morph task. Participants were asked to identify the emotions of 30 facial expressions. This included one 100% version of the angry, sad, fearful, disgusted, surprised and happy faces used in the Emotion Morph task (six images in total), and four 65% versions of these faces (with 35% being another of the six basic emotions). We used 65% because that presented a combination that was more subtle than the 100% versions, but still clearly identifiable as a particular expression. The morphs were included to increase the difficulty level and make the task more sensitive to potential age differences in recognition. Images remained on the screen and participants had an unlimited time to choose from amongst six emotion labels (angry, sad, etc.), with total proportion correct used in the analyses.

#### Eyes task

We gave participants the 36 items of the revised version of the Adult “Reading the Mind in the Eyes” test^70^. Each item included a still black-and-white photograph of the eye region of the face, along with four corresponding words presented on the corners of the screen. Images remained on the screen and participants had an unlimited time to choose the word that best described the person’s feelings or thoughts, with total proportion correct used in the analyses.

#### Ethics

This study was approved by the University Human Ethics Committee (reference number 19/140) and carried out consistent with American Psychological Association ethical guidelines. Prior to beginning, informed consent was obtained from all participants.
